# Staphylococcical scalded skin syndrome: a case series description of a rare and critical disease in a tertiary pediatric center

**DOI:** 10.1186/s13052-025-02078-5

**Published:** 2025-09-02

**Authors:** Agnese Tamborino, Elisabetta Venturini, Carlotta Montagnani, Leila Bianchi, Giuseppe Indolfi, Elena Chiappini, Luisa Galli, Sandra Trapani

**Affiliations:** 1 Infectious Disease Unit, Meyer Children’s Hospital IRCCS, Florence, Italy; 2 Pediatric Unit, Meyer Children’s Hospital IRCCS, Florence, Italy; 3 Department of NEUROFARBA, University of Florence, Florence, Italy; 4 Department of Health Sciences, University of Florence Meyer Children’s Hospital IRCCS, Viale Pieraccini, 24, Florence, 50139 Italy

**Keywords:** Staphylococcal scalded skin syndrome, Antibiotics, Pediatrics, Skin infections, Blisters, Desquamation, Erythroderma, Exfoliative toxins, Children, Staphylococcus aureus

## Abstract

**Background:**

Staphylococcal-scalded skin syndrome (SSSS) is a potentially life-threatening disorder characterized by superficial skin blistering caused by exfoliative toxins produced by *Staphylococcus aureus*. This study aimed to investigate SSSS in a cohort of children admitted at a tertiary pediatric hospital in Italy.

**Methods:**

Patients discharged with the diagnosis of staphylococcal infection and of SSSS between January 2010 and March 2023 were retrospectively identified using ICD-9-CM codes (695.81 and 041.1, respectively). Medical records were reviewed to extract epidemiological, clinical, and hematological data, treatment details (type and duration), length of hospitalization, and outcomes.

**Results:**

Among 971 children with staphylococcal infection, 21 (2.1%) were diagnosed with SSSS. The mean age of 36.8 (interquartile range, IQR 8.5–50.7) months, with 86% under 5 years old. Incidence peaked in winter, summer, and autumn (27.3%, respectively), possibly due to viral co-infection. The admissions/year rate did not indicate an upward trend. Almost all children were healthy. No previous trauma, insect bites, drugs, vaccines, or allergy history have been reported; atopic dermatitis has been reported in one girl. Leukocytosis and elevated C-reactive protein were uncommon. Severe complications were seen in three cases (14.3%): one with severe dehydration with hyponatremia, one with sepsis and the last with Herpes Simplex Virus 1 (HSV1) infection. *S. aureus* was detected by culture from skin lesions in nine cases (42.9%), by real-time polymerase chain reaction (*RT-PCR*) assay on vesicle fluid in seven (33%), and by throat culture in one (4.7%). Drug susceptibility tests ruled out resistance and all children received intravenous (IV) antibiotics: oxacillin in 76% of patients, while teicoplanin and clindamycin in 19%. The median duration of IV and oral antibiotic therapy was 12.8 days (IQR 10–14). Only one patient was treated with IV immunoglobulin. The median hospitalization length was 7.8 days (IQR 5–9). All our cases had a favorable outcome.

**Conclusion:**

Demographic, clinical. and hematological features of children with SSSS in this study were comparable with those reported in the literature. The improved awareness of pediatricians should faster diagnosis, which is mainly clinical, and early assessment of appropriate management.

## Background

Staphylococcal scalded skin syndrome (SSSS) is a potentially life-threatening disorder characterized by erythema and superficial skin blistering [1]. It is a toxin-mediated disease caused by exfoliative toxins A (ETA) and B (ETB) produced by certain strains of *Staphylococcus aureus* [2]. Although these toxins act locally on the skin, they may enter the bloodstream and cause a systemic inflammatory response. Therefore, the cutaneous disease may be accompanied by fever and signs of systemic inflammation, either clinical or biochemical.

The condition was first described by Ritter von Rittershain [3] and the term “staphylococcal scalded skin syndrome” was introduced by Melish and Glasgow nearly a century later [4]. Epidemiological data on SSSS remain scarce: in France and Germany, the incidence reported is 0.09 and 0.56 per-million persons per year, respectively [5, 6]; in the US, the annual incidence is 7.67 (range: 1.83–11.88) per-million children, with 45.1 cases per-million infants under 2 years of age [7]. African–American children are less susceptible than Caucasian ones [8, 9]. The condition is most frequently observed in neonates and children under 5 years, with the highest incidence occurring between 2 and 3 years old [1, 10–12]. SSSS can also occur in older children and adults with renal failure, immunologic deficiency, and other chronic illnesses [13–16]. The higher risk in children, especially neonates, may be related to their immature immune system and renal clearance ability [17]. The morbidity rate in young children is high, primarily due to complications such as secondary infections, hypothermia, and dehydration resulting from fluid loss through damaged skin with the consequent electrolyte imbalances [1].

In contrast, the overall mortality rate for SSSS in children is quite low, ranging from 0.3% [18] to 5% [19], unless associated with sepsis or a severe underlying disease.

While most SSSS cases are attributed to methicillin-sensitive *Staphylococcus aureus (MSSA)*, an increase in methicillin-resistant *Staphylococcus aureus* (*MRSA*) has also been recently observed [20, 21]. The syndrome presents as a macular erythema followed by bullous lesions and diffuse epidermal exfoliation. A prodromal localized staphylococcal infection of the skin, throat, nose, mouth, umbilicus, or gastrointestinal tract typically occurs. General malaise, fever, irritability, skin tenderness, and reduced appetite may also be present. Other signs, such as facial oedema, conjunctivitis, and perioral crusting, can also be present. Mucous membranes are typically spared, but dehydration may be a serious complication. Gentle pressure on apparently normal skin results in the separation of the upper epidermis (Nikolsky’s sign) [7]. Complications of SSSS include secondary viral (herpes viruses) or bacterial infections (cellulitis, sepsis, and pneumonia), dehydration, electrolyte imbalance, and hypothermia [22, 23]. Management involves administering anti-staphylococcal antibiotics, fluids, and electrolytes, as well as topically treating the denudated areas [1]. In this study, we aimed to investigate the clinical spectrum of SSSS in a cohort of children admitted to a tertiary pediatric hospital in Italy.

## Methods

### Study design and population

We retrospectively identified children (aged 0–14 years) with evidence of Staphylococcal infection who were admitted at Meyer Children’s Hospital IRCCS, from January 2010 to March 2023.

### Inclusion and exclusion criteria

We identified discharges with either a principal or secondary diagnosis of Staphylococcal infection using the International Classification of Disease, Ninth Revision, Clinical Modification (ICD-9-CM) code 041.1. Among these, we selected children with a final diagnosis of SSSS (code 695.81). Exclusion criteria were as follows: localized bullous impetigo (BI), bacteremia, endocarditis, osteomyelitis, pneumonia, and septic arthritis.

### Data collection

Medical records of the included patients were reviewed to collect the following data:

- demographic data: gender, age, geographic area, and season at onset;
- historical data: familial and personal history, including previous diseases/comorbidities, sport activity and previous hospital admissions;
- clinical features: cutaneous signs, involved areas, Nikolsky’s sign positivity, and status of desquamation, as well as systemic symptoms such as fever, pain, weakness, irritability, and loss of appetite;
- clinical types: based on Kang et al. [24], we classified cases into three types: the generalized type was defined as a large area of skin lesions (over 30% of the whole body) with tender erythroderma, large bullae, and a positive Nikolsky’s sign; the intermediate type was defined as skin lesions with tender erythroderma, vesicles, or pustules in a regionally limited area (< 30%) and a positive Nikolsky’s sign; the abortive type was defined in case of diffuse scarlatiniform rash with tender erythema without Nikolsky’s sign [24];
- complications: severe dehydration with electrolyte imbalance, sepsis, and secondary infections;
- laboratory investigations: C-reactive protein (CRP), white blood cell (WBC) count, procalcitonin (PCT), electrolytes, and immunological parameters (immunoglobulins, dihydrorhodamine testing and lymphocyte subsets), when available;
- microbiological data: detection of *S.aureus* by both standard culture methods and real-time polymerase chain reaction (RT-PCR) assays;
- management: local and systemic therapies (type and duration);
- outcome: length of hospital stay (LOS) and the number of readmissions within two weeks following discharge.

### Ethical considerations

The study protocol adhered to the ethical guidelines of the 1975 Declaration of Helsinki (6th version, 2008) and was approved by the Institutional Review Board of Meyer Children’s Hospital IRCCS.

### Statistical analysis

Descriptive statistics (means, IQRs, percentages) were performed to analyse continuous and categorical data. We conducted a univariate logistic regression analysis to assess potential associations between demographic and clinical variables with both the duration of therapy and the length of hospitalization.

## Results

Nine hundred seventy-one children with Staphylococcal infection were identified. Twenty-one discharged with the diagnosis of SSSS (21/971, 2.1%) were selected (Table 1). Median age was 36.8 (IQR 8.5–50.7) months, and 86% of children were under 5 years old. Eleven were males (52,4%). Most patients (90.5%) had no underlying condition identified; one patient had a pre-existing cutaneous disease (atopic dermatitis), and one suffered from phenylketonuria. No history of previous trauma, insect bites, drugs, vaccines, or allergy was reported; six children participated in sporting activities. None of the patients had a previous episode of SSSS, or a positive family history of cutaneous diseases. The incidence of SSSS varied by season, with the highest rates occurring in autumn, winter, and summer (27.3%, each). Eight children (38%) presented with respiratory symptoms upon admission. The admissions were evenly distributed throughout the study period (2 cases per year). All patients were admitted to the Pediatric Ward; none required escalation of care to intensive care unit (ICU), and no deaths were reported.

**Table 1:** Clinical characteristics of 21 children with SSSS

Characteristic		N of cases	%
*Demographic data*	Male Sex	11	52.4
	Mean age (range), months	36.8 (IQR 8.5–50.7)	
	Season: Autumn	6	27.3
	Winter	6	27.3
	Spring	3	18.1
	Summer	6	27.3
*Systemic signs/symptoms*	Low appetite	12	57
	Pain	12	57
	Fever	7	33
	Irritability	6	28.6
*Clinical signs*	Skin erythema	21	100
	Exfoliation/desquamation	21	100
	Skin tenderness	15	71.4
	Facial edema	3	14.2
	Perioral crusting	11	52.4
	Vesicles/ bullae	6	28.6
	Periocular crusting	11	52.4
	Positive Nikolsky's sign	20	95.2
	Perinasal crusting	10	47.6
	Conjunctivitis	8	38
	Mucous membrane involvement	2	9.5
*Source of infection*	Nares and/or throat	1	4.8
	Skin and/or soft tissue	8	38
	Not reported	12	57
*Complications*	Sepsis	1	4.8
	HSV1 infection	1	4.8
	severe dehydration	1	4.8
*Outcome*	Healing	21	100
	Re-admissions	0	0
	LOS, days	7.8 (IQR 5–9)	

**Fig. 1:**
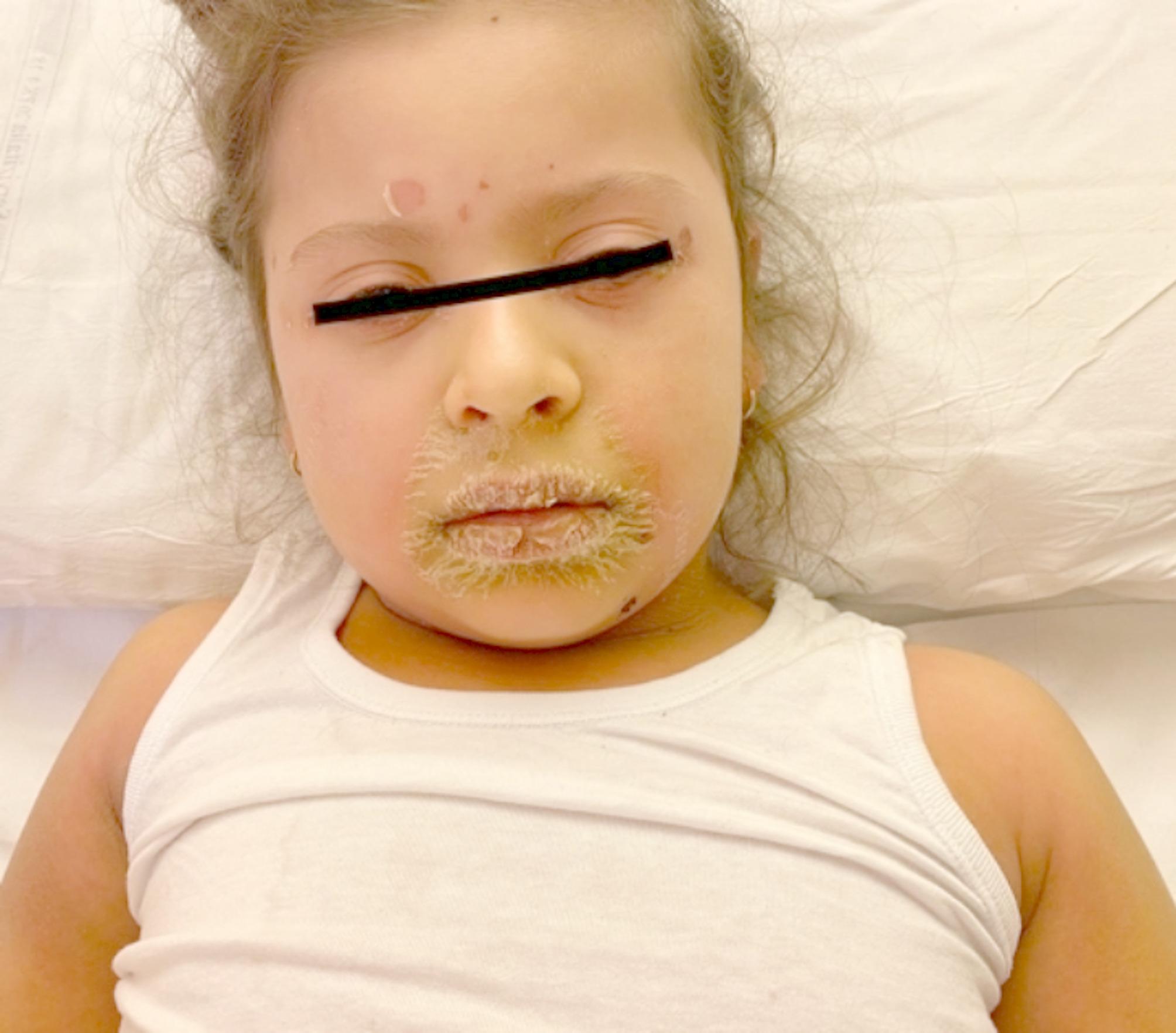
Diffuse erythroderma and desquamation in a child with staphylococcal scalded skin syndrome on day 3 after the onset

Physical examination revealed varying degrees of cutaneous erythema and superficial desquamation with periorificial, flexural, and/or acral accentuation in all patients. According to Kang’s classification [27], the generalized type (Fig. 1) was observed in 13 patients (62.5%), the intermediate in seven (33%), and the abortive type in one (4.5%). Other involvement was noted in eight children: conjunctivitis in seven patients, and oral mucosa involvement in two cases. Systemic symptoms included pain in 11 cases (50%), fever in eight (38%), irritability in six (28%), low appetite in 12 (57%), and pruritus in eight patients (38%).

The diagnosis of SSSS was clinically confirmed in all patients and by the dermatologist in ten cases. One young girl had a skin biopsy performed at local hospital which revealed necrotic keratinocytes with early bullae formation, initially leading to the diagnosis of erythema multiforme. Direct immunofluorescence showed linear deposition of immunoglobulin M and C_3_ complement at the dermo-epidermal junction. Upon transfer to our hospital, the physical examination revealed diffuse skin exfoliation. Clinical assessment by a burn surgeon, dermatologist, and ophthalmologist confirmed the final diagnosis of SSSS, excluding mucosal involvement.

Regarding the laboratory tests, high CRP levels and leucocytosis were observed in 28.6% of cases, eosinophilia in 9.5%, and PCT was always normal. Electrolytes were normal in all patients except one, who had hyponatremic dehydration.

Serum immunoglobulin profile, dihydrorhodamine testing, and lymphocyte subsets, performed in 11 cases, were normal. One patient has antinuclear antibodies (ANA) positivity with a title 1:160, dense, fine speckled pattern. Coeliac disease antibodies were tested in one patient and were negative.

Microbiological findings revealed *S. aureus* in 61.9% of cases. *S. aureus* was detected in cultures from periorificial lesions in nine cases (42.9%), by *RT-PCR* assay on vesicle fluid in seven (33%), and by throat culture in one (4.7%). Panton-Valentine Leucocidin (PVL) was tested in four children and was negative in all cases. No testing for exfoliating toxins was performed in our laboratory.

Sensitivity data for *S. aureus* were available for 10 patients (47%). No isolates were resistant to methicillin, vancomycin, or fusidic acid. Resistance to clindamycin and erythromycin was observed in two isolates.

Five patients had other pathogens isolated by cultures from skin lesions: co-pathogens included *Enterococcus faecalis* in three cases, *Moraxella catarrhalis* in one, and *Streptococcus mitis* associated with *Actinomyces* in another one. In the latter patient, MSSA, *S. mitis*, and *Actinomyces* were cultured also from wound swabs. Blood cultures, obtained from five patients, were negative.

A quantitative *RT-PCR* test on the bloodstream was positive for Human Herpesvirus (HHV) 7 in two patients, HHV 6 in one, Herpes simplex virus 1 (HSV1) in one, and *Escherichia coli* in another child.

Severe complications were seen only in three cases (14.3%): one girl presented severe dehydration with hyponatremia, one had sepsis, and the last had severe HSV1 infection.

All children received IV antibiotics during hospitalization. Only one patient was treated with IV immunoglobulin (1 g/kg). The empiric antimicrobial treatment was oxacillin as the first-line drug in 16 patients, either alone or as part of a combined treatment. Four patients were given clindamycin alone or in combination with other antibiotics, and one patient was given ceftriaxone. The target antimicrobial treatment was oxacillin alone for 10 patients, oxacillin plus another drug (ceftriaxone or clindamycin) for six patients, teicoplanin alone for three patients, and teicoplanin with clindamycin for two cases. Five patients completed the intravenous antibiotic regimen entirely in the hospital. In the remaining 16 cases, therapy was switched to oral antibiotics, including amoxicillin-clavulanate (11 cases), cefixime (1 case), cephalexin (1 case), trimethoprim-sulfamethoxazole combined with rifampicin (1 case), clindamycin plus cefixime (1 case), and amoxicillin-clavulanate combined with rifampicin (1 case). The median length of oral antibiotics was 5.8 days (IQR 5–7). The median duration of IV plus oral antibiotic therapy was 12.8 days (IQR 10–14). In addition to systemic antibiotics, a local treatment with emollients, fusidic acid or mupirocin, eye drops, pain medications, such as acetaminophen, and antihistamine drugs, such as cetirizine or oxatomide, were also prescribed in 76% of cases. All of them received IV fluids. None required enteral tube feeding. No surgical debridement of the skin has been performed.

The median LOS was 7.8 days (IQR 5–9) with a slight difference between boys and girls: the average LOS was 8.2 days for the boys and 7.2 days for the girls.

In the univariate logistic regression (Table 2), none of the assessed variables showed statistically significant associations with the duration of therapy. Although male sex and the presence of siblings appeared to be linked to a longer duration of treatment, these findings did not reach statistical significance. Regarding the univariate analysis for factors associated with the length of hospitalization (Table 3), none of the variable analysed were significantly associated with a longer stay.

**Table 2:** Univariate analysis of factors associated with duration of therapy

	UNIVARIATE ANALYSIS		
	uOR	CI 95%	*p*
*Gender*	2,473	0,419; 16,664	0,3949
*Age (months)*	0,576	0,090; 3,382	0,6699
*Community exposure*	1,135	0,149; 8,673	1
*Siblings*	2,62	0,433; 19,045	0,387
*Tmax (°C)*	1,187	0,200; 7,204	1
*Season*	1,692	0,283; 10,904	0,6699
*Previous hospitalizations*	1,118	0,097; 12,916	1
*Sports*	0,881	0,115; 6,701	1

**Table 3:** Univariate analysis of factors associated with length of stay

	UNIVARIATE ANALYSIS		
	uOR	CI 95%	*P*
*Gender*	2,772	0,380; 28,366	0,3615
*Age (months)*	1,135	0,149; 8,673	1
*Community exposure*	0,449	0,014; 4,183	0,6227
*Siblings*	0,681	0,088; 5,202	1
*Tmax (°C)*	2,772	0,380; 28,366	0,3615
*Season*	3,63	0,491; 37,847	0,331
*Sports*	0,727	0,088; 7,707	1

All our patients, including those with complications, healed without sequelae and none was re-admitted to our hospital in the following two weeks.

## Discussion

*S. aureus* causes a variety of infectious diseases, ranging from superficial skin infections to severe, toxin-mediated systemic infections. SSSS is a type of systemic disorder mainly caused by coagulase-positive group II *S. aureus* that can cause superficial skin blistering. Skin acantholysis leads to cracks in the epidermis and characteristic skin bullae [25]. The pathophysiologic mechanisms include several steps: first, the production of exfoliative toxins (ETs). Several strains of *Staphylococcus aureus* (Group II) can produce epidermolytic toxins, primarily toxin A (ETA) and toxin B (ETB), which are exfoliative. These toxins have a specific affinity for skin proteins, particularly a skin protein called desmoglein-1 (Dsg-1), a desmosomal cadherin of the superficial epidermis responsible for the adhesion of skin cells (the upper layer of the skin). Thus, the epidermolytic toxins act by binding to Dsg-1 and causing cleavage of the desmosomes, leading to separation between the epidermal cells of the skin. Following the disruption of the desmosomes, epidermal cells detach from one another, causing the formation of blisters, bullae, and peeling of the skin. This process leads to the typical manifestation of skin exfoliation, which may appear like a burn [26].

The diagnosis is clinical and the consultation with a dermatologist should be considered if the diagnosis is uncertain. SSSS may resemble other exfoliative skin conditions, such as toxic epidermal necrolysis (TEN), epidermolysis bullosa (EB), Stevens–Johnson syndrome (SJS), toxic shock syndrome (TSS), and pemphigus. Differentiation is primarily based on the presence of mucosal involvement. Bullous impetigo, caused by the localized release of similar toxins, often presents with similar skin findings but is typically confined to specific areas, while SSSS causes a more widespread rash and severe symptoms due to the systemic distribution of these toxins. These conditions may occasionally be mistaken for other disorders that result in superficial blistering [27].

Our retrospective study analysed 21 cases of SSSS from 2010 to 2023 in a tertiary care Children’s Hospital IRCCS. SSSS commonly affects neonates and children younger than 5 years, with peak incidence between 2 and 3 years. It is well established that neonates and young children are at greater risk due to their immature immune system, which is unable to neutralize epidermolytic toxins and their limited renal capacity to eliminate these toxins [28, 29]. Similarly, immunocompromised adults or those with renal impairment present a higher incidence [30].

The increased frequency and severity of SSSS in neonates likely results from a combination of factors, including the reduced renal clearance of toxins and the underdeveloped structure of the neonatal epidermis. In addition to age, other predisposing conditions are recognized risk factors for SSSS, particularly in severe cases. These include atopic dermatitis, renal failure, and primary immunodeficiencies [26]. For instance, Hyper-IgE syndrome [31] and Netherton syndrome [32] have been described in children with severe SSSS. In our cohort, none of the patients presented with predisposing conditions, except one child with atopic dermatitis.

A large U.S. study on 589 children with SSSS reported an increase in incidence between 2010 and 2012 compared to 2008–2009 [7]. Similarly, Li et al. observed a rise in incidence Chinese infants, with 79,5% occurring between 2008 and 12 compared to 20,5% between 2004 and 2009 [30]. This upward trend aligns with other previous reports [33, 34]. Conversely, in Europe and regions with high hygiene standards, a decline in SSSS incidence has been noted [6]. In our study, hospital admissions remained stable over time, with an average of two cases per year.

Consistent with the literature [1, 11, 12], most of our patients (86%) were under 5 years years of age, with a slight male predominance as reported in other studies [28, 35]. However, Lyi-Wong [35], found no sex predominance.

Seasonal distribution in our series showed that SSSS was more frequent during winter, autumn, and summer, in line with prior studies [33, 35–37]. Some authors suggest that viral upper respiratory tract (URT) infections, particularly in autumn, may predispose colonized individuals to SSSS [5]. Previous studies have also documented preceding infections such as URT infections otitis media, conjunctivitis, omphalitis [30], pneumonia [33], pyomyositis [38], and maternal breast abscesses [39]. Similarly, we observed that eight children presented URT symptoms prior to hospitalization.

The clinical course of SSSS is typically characterized by a prodromal phase with fever, irritability, and anorexia. Before the onset of skin lesions, Nikolsky’s sign may already be positive. Initial dermatologic findings include widespread erythematous patches and the formation of fragile bullae, which rupture easily, leaving behind denuded, scalded-appearing skin [28, 29, 40]. Exfoliated areas crust over within 24 h, with perioral and periorbital fissures, and complete re-epithelialization generally occurs within two weeks without scarring [4]. In our series, these features were variably present and summarized in Table 1. Based on Kang et al.’s classification [24], the generalized type was most frequent (62.5%), followed by the intermediate type, while the abortive form was rare. Systemic symptoms such as poor appetite and pain were common, while fever and irritability were present in approximately one-third of cases.

In our series, complications occurred in 14.2% of patients and included sepsis, dehydration with hyponatremia, and HSV1 infection. Literature reports that SSSS severity is influenced by toxin burden and the host’s immune response. The compromised skin barrier predisposes to secondary infections, hypothermia, and fluid loss. Hence, dehydration and electrolyte imbalances, notably hyponatremia, are frequent complications, underscoring the need for careful fluid management and laboratory monitoring. Interestingly, Blyth et al. reported that fluid overload can paradoxically occur despite hypovolemia being the primary concern [41].

Although rare, other complications include pneumonia and bacteremia (sepsis). Life-threatening events such as toxic shock syndrome [35, 36], acute kidney injury, and thrombosis have also been reported [42].

Regarding laboratory findings, leukocytosis and elevated CRP levels were detected in only 28.6% of cases, indicating a mild or delayed systemic inflammatory response. Consistent with Neubauer et al., laboratory tests (WBC, ESR, CRP) showed limited prognostic value in SSSS management [36]. Zeng et al. demonstrated elevated WBC, CRP, IL-6, and PCT in both bacterial infections and SSSS compared to healthy controls, with PCT levels being notably higher in SSSS [40]. However, in our series, PCT remained negative in all patients, even at onset.

The detection rate of *S.aureus* was high (62%) in our study, which is consistent with prior reports [28, 43]. Blood cultures yielded no significant results, in line with the low positivity rate typically seen in pediatric populations [5, 33, 35, 44–46] in contrast to adult data [26].

Early diagnosis and timely administration of appropriate parenteral anti-staphylococcal antibiotics, along with supportive care, are crucial. Empirical therapy in our cohort mainly involved oxacillin, alone or in combination, reflecting current recommendations [25, 39]. The ten positive cultures (nine from skin lesions and one from throat swab) all grew MSSA.

In settings with high MRSA prevalence, vancomycin or teicoplanin should be considered upfront, particularly in severe or non-responsive cases [47]. Although no MRSA strains were isolated, six patients required teicoplanin following oxacillin failure, three of whom were critically ill. Additionally, four patients were treated with clindamycin, alone or in combination.

Despite increasing reports of clindamycin resistance [48], resistance was identified in only two isolates (10%) in our study. In contrast, Braunstein et al. reported a clindamycin resistance rate of 52% despite MSSA isolates [47]. Wang et al. also found SSSS-associated strains more frequently resistant to clindamycin but less often resistant to methicillin compared to other S. aureus infections [48].

All patients in our series survived, including those with complications, which aligns with previous studies [35]. This contrasts with larger studies reporting pediatric mortality rates between 0.3% and 5% [4, 6, 40]. In adults, mortality is significantly higher (40-63%) due to underlying comorbidities [28]. Fatal outcomes in children are primarily linked to severe complications such as pneumonia and sepsis [48].

In our cohort, the median length of stay (LOS) was 7.8 days, which is comparable to other studies reporting LOS ranging from 3 to 8 days [35, 48], and even longer among neonates [45]. No readmissions were recorded, consistent with the literature describing recurrence as exceedingly rare [10].

This case series provides insight into the clinical features, management, and outcomes of pediatric SSSS. Early diagnosis and intervention are paramount to reducing morbidity and mortality. Our data suggest a stable incidence in our setting, but results may differ in other geographic areas.

Study limitations include its retrospective design, incomplete data, small sample size, and single-center setting. Furthemore, we lacked data on S. aureus phage types and toxin profiles, limiting etiological characterization. Future studies should be prospective, multicenter investigations incorporating toxin profiling and resistance trends, as well as long-term follow-up to assess recurrence and late sequelae.

## Data Availability

The datasets generated during and/or analysed during the current study are not publicly available but are available from the corresponding author on reasonable request.

## Abbreviations

SSSS: Staphylococcal-scalded skin syndrome
IQR: Interquartile range
RT-PCR: Real- time polymerase chain reaction
IV: Intravenous
MSSA: Methicillin-sensitive Staphylococcus aureus
MRSA: Methicillin-resistant Staphylococcus aureus
BI: Bullous impetigo
CRP: C-reactive protein
WBC: White blood cells
PCT: Procalcitonin
LOS: Length of stay
ICU: Intensive care unit
ANA: Antinuclear antibodies
PVL: Panton valentine leucocidin
URT: Upper respiratory tract
HSV1: Herpes simplex virus 1

## References

[1] Mishra AK, Yadav P, Mishra A. A systemic review on Staphylococcal scalded skin syndrome (SSSS): A rare and critical disease of neonates. Open Microbiol J. 2016;10:150–9. 27651848 10.2174/1874285801610010150 PMC5012080

[2] Ladhani S. Understanding the mechanism of action of the exfoliative toxins of Staphylococcus aureus. FEMS Immunol Med Microbiol. 2003;39:181–9. 14625102 10.1016/S0928-8244(03)00225-6

[3] Ritter von Rittershain G. Die exfoliativa dermatitis Jungerer Saulinge. Centralz Kinderheilk. 1878;2:3–23.

[4] Melish ME, Glasgow LA. The Staphylococcal scalded skin syndrome. N Engl Med. 1970;282:1114–9. 10.1056/NEJM197005142822002 4245327

[5] Lamand V, Dauwalder O, Tristan A, Casalegno JS, Meugnier H, Bes M, et al. Epidemiological data of Staphylococcal scalded skin syndrome in France from 1997 to 2007 and Microbiological characteristics of *Staphylococcus aureus* associated strains. Clin Microbiol Infect. 2012;18:e514–21. 23078129 10.1111/1469-0691.12053

[6] Mockenhaupt M, Idzko M, Grosber M, Schöpf E, Norgauer J. Epidemiology of Staphylococcal scalded skin syndrome in Germany. J Invest Dermatol. 2005;124:700–3. 15816826 10.1111/j.0022-202X.2005.23642.x

[7] Staiman A, Hsu DY. Silverberg. Epidemiology of Staphylococcal scalded skin syndrome in U.S. Children. Br J Dermatol. 2018;178(3):704–8. 29077993 10.1111/bjd.16097

[8] Berk D. Staphylococcal scalded skin syndrome. Clinical decision support: pediatrics. Wilmington, delaware: decision support in medicine, LLC., 2015, electronic database. http://www.decisionsupportinmedicine.com. Accessed 13 Nov 2017.

[9] Manders SM et al. In: Heyman WR, Anderson BE, Hivnor C, editors. Clinical Decision Support: Dermatology. Wilmington, Delaware: Decision Support in Medicine, LLC, 2015, 2nd edition, electronic database. http://www.decisionsupportinmedicine.com. Accessed 14 Nov 2017.

[10] Davidson J, Polly S, Hayes PJ, Fisher KR, Talati AJ, Patel T. Recurrent Staphylococcal scalded skin syndrome in an extremely low-birth-weight neonate. AJP Rep. 2017;7:e134–7. 28674637 10.1055/s-0037-1603971 PMC5493488

[11] Arora P, Kalra VK, Rane S, McGrath EJ, Zegarra-Linares R, Chawla S. Staphylococcal scalded skin syndrome in a preterm newborn presenting within first 24 h of life. BMJ Case Rep. 2011;2011:bcr0820114733. 22670002 10.1136/bcr.08.2011.4733 PMC3246154

[12] Oliveira AR, Aires S, Faria C, Santos E. Staphylococcal scalded skin syndrome. BMJ Case Rep. 2013; 2013: bcr2013009478. 10.1136/bcr-2013-009478 PMC3703018 23761500

[13] Saida K, Kawasaki K, Hirabayashi K et al. Exfoliative toxin A Staphylococcal scalded skin syndrome in preterm infants. Eur J Pediatr. 2015 (174):551–5. 10.1007/s00431-014-2414-3 25194957

[14] Blyth M, Estela C, Young AE, et al. Severe Staphylococcal scalded syndrome in children. Burns. 2008;34:98–103. 17644261 10.1016/j.burns.2007.02.006

[15] Rydzewska-Rosolowska A, Brzosko S, Borawski J, Mysliwiec M. Staphylococcal scalded skin syndrome in the course of lupus nephritis. Nephrol (Carlton). 2008;13:265–66. 10.1111/j.1440-1797.2007.00904.x 18315709

[16] Yamasaki O, Takayuki Y, Motoyuki S et al. Clinical manifestations of Staphylococcal Scalded-Skin syndrome depend on serotypes of exfoliative toxins. J Clin Microbiol. 2005(43):1890–3. 10.1128/JCM.43.4.1890-1893.2005 PMC1081326 15815014

[17] Norbury WB, Gallagher JJ, Herndon DN, Branski LK, Oehring PE, Jeschke MG. Neonate twin with Staphylococcal scalded skin syndrome from a renal source. Pediatr Crit Care Med. 2010;11:e20–3. 20216172 10.1097/PCC.0b013e3181b80dd2

[18] Arnold JD, Hoek SN, Kirkorian AY. Epidemiology of Staphylococcal scalded skin syndrome in the united states: a cross-sectional study, 2010–2014. J Am Acad Dermatol. 2018;78:404–6. 29332709 10.1016/j.jaad.2017.09.023

[19] Jordan KS. Staphylococcal scalded skin syndrome: A pediatric dermatological emergency. Adv Emerg Nurs J. 2019. 10.1097/TME.0000000000000235 31033660

[20] Liassine N, Auckenthaler R, Descombes MC, et al. Community-acquired methicillin- resistant Staphylococcus aureus isolated in Switzerland contains the Panton-Valentine leukocidin or exfoliative toxin genes. J Clin Microbiol. 2004;42:825–8. 14766862 10.1128/JCM.42.2.825-828.2004 PMC344453

[21] Noguchi N, Nakaminami H, Nishijima S, et al. Antimicrobial agent of susceptibilities and antiseptic resistance gene distribution among methicillin-resistant Staphylococcus aureus isolates from patients with impetigo and Staphylococcal scalded skin syndrome. J Clin Microbiol. 2006;44:2119–25. 16757607 10.1128/JCM.02690-05 PMC1489400

[22] Aydin D, Alsbjørn B. Severe case of Staphylococcal scalded skin syndrome in a 5-year-old child—case report. Clin Case Rep. 2016;4:416–9. 27099742 10.1002/ccr3.535 PMC4831398

[23] Berk DR, Bayliss SJ. MRSA, Staphylococcal scalded skin syn- drome, and other cutaneous bacterial emergencies. Pediatr Ann. 2010;39:627–33. 20954609 10.3928/00904481-20100922-02

[24] Nishifuji K, Shimizu A, Ishiko A, et al. Removal of amino-terminal extracellular domains of Desmoglein 1 by Staphylococcal exfoliative toxin is sufficient to initiate epidermal blister formation. J Dermatol Sci. 2010;59:184–91. 20728315 10.1016/j.jdermsci.2010.07.010

[25] Handler MZ, Schwartz RA. Staphylococcal scalded skin syndrome: diagnosis and management in children and adults. J Eur Acad Dermatol Venereol. 2014;28:1418–23. 24841497 10.1111/jdv.12541

[26] Rouva G, Vergadi E, Krasagakis K, Galanakis E. Understanding host’s response to Staphylococcal scalded skin syndrome. Acta Paediatr. 2025;114(2):241–7. 39411997 10.1111/apa.17462 PMC11706759

[27] Brazel M, Desai A, Are A, Motaparthi K. Staphylococcal scalded skin syndrome and bullous impetigo. Med (Kaunas). 2021;57(11):1157. 10.3390/medicina57111157 PMC8623226 34833375

[28] Ladhani S. Recent developments in Staphylococcal scalded skin syndrome. Clin Microbiol Infect. 2001;7:301–7. 11442563 10.1046/j.1198-743x.2001.00258.x

[29] Amagai M, Matsuyosih N, Wang ZH, et al. Toxin in bullous impetigo and Staphylococcal scalded-skin syndrome targets Desmoglein 1. Nat Med. 2000;6:1275–7. 11062541 10.1038/81385

[30] Li MY, Hua Y, Wei GH, et al. Staphylococcal scalded skin syndrome in neonates: an 8-year retrospective study in a single institution. Pediatr Dermatol. 2014;31:43–7. 23557104 10.1111/pde.12114

[31] Ajmi H, Jemmali N, Mabrouk S, Hassayoun S, Ben-Ali M, Barbouche MR, Mokni M, Abroug S. Staphylococcal scalded skin syndrome: an uncommon symptomatology revealing an immune deficiency. Arch Pediatr. 2018;25(2):126–8. 29248323 10.1016/j.arcped.2017.11.008

[32] Chao SC, Richard G, Lee JY. Netherton syndrome: report of two Taiwanese siblings with Staphylococcal scalded skin syndrome and mutation of SPINK5. Br J Dermatol. 2005;152(1):159–65. 15656819 10.1111/j.1365-2133.2005.06337.x

[33] El Helali N, Carbonne A, Naas T, et al. Nosocomial outbreak of Staphylococcal scalded skin syndrome in neonates: epidemiological investigation and control. J Hosp Infect. 2005;61:130–8. 16009455 10.1016/j.jhin.2005.02.013

[34] Lipovy B, Brychta P, Chaloupkova Z, et al. Staphylococcal scalded skin syndrome in the Czech republic: an epidemiological study. Burns. 2012;38:296–300. 22035884 10.1016/j.burns.2011.08.005

[35] Liy-Wong C, Pope E, Weinstein M, Lara-Corrales I. Staphylococcal scalded skin syndrome: an epidemiological and clinical review of 84 cases. Pediatr Dermatol. 2021;38(1):149–53. 33283348 10.1111/pde.14470

[36] Hannah C, Neubauer M, Hall, Sowdhamini S, Wallace, et al. Variation in diagnostic test use and associated outcomes in Staphylococcal scalded skin syndrome at children’s hospitals. Hosp Pediatr. 2018;8(9):530–7. 30139766 10.1542/hpeds.2018-0032 PMC6317540

[37] Wong GW, Oppenheimer SJ, Evans RM, et al. Pyomyositis and Staphylococcal scalded skin syndrome. Acta Paediatr. 1993;82:113–5. 8453207 10.1111/j.1651-2227.1993.tb12536.x

[38] Raymond J, Bingen E, Brahimi N, et al. Staphylococcal scalded skin syndrome in a neonate. Eur J Clin Microbiol Infect Dis. 1997;16:453–4. 9248748 10.1007/BF02471909

[39] Patel GK, Finlay AY. Staphylococcal scalded skin syn- drome: diagnosis and management. Am J Clin Dermatol. 2003;4:165–75. 12627992 10.2165/00128071-200304030-00003

[40] Zeng M, Guo Z, Shen S, Liu S. Value of serum procalcitonin and interleukin-6 in patients with bullous impetigo and Staphylococcal scalded skin syndrome. J Dermatol. 2014;41(11):1028–9. 25298068 10.1111/1346-8138.12638

[41] Blyth M, Estela C, Young AE. Severe Staphylococcal scalded skin syndrome in children. Burns. 2008;34(1):98–103. 17644261 10.1016/j.burns.2007.02.006

[42] Keating M, Yoo LJH, Lane-O’Neill B, Moran T, Ni Ainle F, Moloney FJ, Potter S. Staphylococcus scalded skin Syndrome-Induced thrombosis leading to free flap complications: A case report and review. Cureus. 2024;16(4):e58173. 38741872 10.7759/cureus.58173 PMC11089487

[43] Chi CY, Wang SM, Lin HC, Liu CC. A clinical and Microbiological comparison of *Staphylococcus aureus* toxic shock and scalded skin syndromes in children. Clin Infect Dis. 2006;42(2):181–5. 16355327 10.1086/498901

[44] Neylon O, O’Connell NH, Slevin B, et al. Neonatal Staphylococcal scalded skin syndrome: clinical and outbreak containment review. Eur J Pediatr. 2010;169(12):1503–9. 20625909 10.1007/s00431-010-1252-1

[45] Paranthaman K, Bentley A, Milne LM, et al. Nosocomial outbreak of staphyloccocal scalded skin syndrome in neonates in England December 2012 to March 2013. Euro Surveill. 2014;19(33):e20880. 10.2807/1560-7917.es2014.19.33.20880 25166346

[46] Iguchi A et al. Vancomycin for severe Staphylococcal scalded skin syndrome. J Paediatr Child Health. 2020. 10.1111/jpc.15027 32596982

[47] Braunstein I, Wanat KA, Abuabara K, McGowan KL, Yan AC, Treat JR. Antibiotic sensitivity and resistance patterns in pediatric Staphylococcal scalded skin syndrome. Pediatr Dermatol. 2014;31(3):305–8. 24033633 10.1111/pde.12195 PMC4349361

[48] Wang Z, Feig JL, Mannschreck DB, Cohen BA. Antibiotic sensitivity and clinical outcomes in Staphylococcal scalded skin syndrome. Pediatr Dermatol. 2020;37(1):222–3. 31626359 10.1111/pde.14014

